# Epstein–Barr virus shapes intrathecal Th1/Th17 immunity and parenchymal involvement in neuro-Behçet’s disease

**DOI:** 10.3389/fimmu.2026.1921667

**Published:** 2026-10-08

**Authors:** Rafika Ben Laamari, Zakaria Saied, Olfa Maghrebi, Khadija Bahrini, Jihène Bekir, Amani Stiti, Adel Driss, Dorra Rezig, Mohamed Ridha Barbouche, Samia Ben Sassi, Meriam Belghith

**Affiliations:** 1 “Transmission, Control and Immunobiology of Infections” Laboratory, Institut Pasteur de Tunis, Tunis, Tunisia; 2 Faculty of Sciences of Tunis, Tunis El Manar University, Tunis, Tunisia; 3 Neurology Department, Mongi Ben Hmida National Institute of Neurology, Tunis, Tunisia; 4 Faculty of Medicine, Tunis El Manar University, Tunis, Tunisia; 5 Department of Experimental and Clinical Medicine (DMSC), University of Florence, Florence, Italy; 6 Department of Physiology, Morehouse School of Medicine, Atlanta, GA, United States; 7 “Virus, Vectors and Hosts” Laboratory, Institut Pasteur de Tunis, Tunis, Tunisia; 8 Department of Microbiology, Immunology and Infectious Diseases, College of Medicine and Health Science, Manama, Bahrain

**Keywords:** cerebrospinal fluid, Epstein-Barr virus, herpesvirus, multiple sclerosis, neuro-Behçet’s disease, neuroinflammation, viral latency

## Abstract

**Introduction:**

Human herpesviruses (HHVs) are implicated in neuro-inflammatory diseases due to their ability to establish persistent latency and reactivate in the context of immune dysregulation. Whilst Epstein -Barr virus (EBV) is recognized as a key factor in multiple sclerosis (MS), its role in neuro-Behçet's disease (NBD) remains poorly understood.

**Methods:**

Serum and paired CSF samples were obtained from treatment-naïve patients with NBD and MS. Neurotropic viral DNA in CSF was screened by multiplex PCR. Virus-specific IgG antibodies against HSV-1 and EBV antigens (VCA and EBNA1) were measured by ELISA in serum and CSF. Real-time PCR was used to assess the expression of EBV latent genes (LMP1 and EBNA1), as well as cytokines and transcription factors, in CSF samples from NBD patients.

**Results:**

In this study, we demonstrate a high prevalence of EBV DNA in the cerebrospinal fluid (CSF) of patients with NBD (71.43%), significantly higher than that observed in MS (35.29%), whereas other neurotropic herpesviruses are rarely detected. Notably, the presence of EBV in the CSF is not associated with a specific intrathecal humoral response, suggesting predominantly latent viral persistence and cell-mediated immune control. The concurrent expression of the latent proteins LMP1 and EBNA1 indicates a type II latency program, capable of activating interferon pathways. EBV-positive patients exhibit an immune signature characterized by significantly elevated levels of IFN-γ and IFN-α, associated with Th1/Th17 polarization and reduced FOXP3 expression, reflecting a Th17/Treg imbalance. Furthermore, EBV status is associated with distinct clinical phenotypes, with parenchymal forms being observed exclusively in EBV-positive patients.

**Conclusion:**

This suggests that EBV may contribute to the pathophysiology of NBD by promoting a pro-inflammatory intrathecal microenvironment. It also positions EBV as a potential biomarker and therapeutic target in this condition.

## Graphical Abstract

**Figure:**
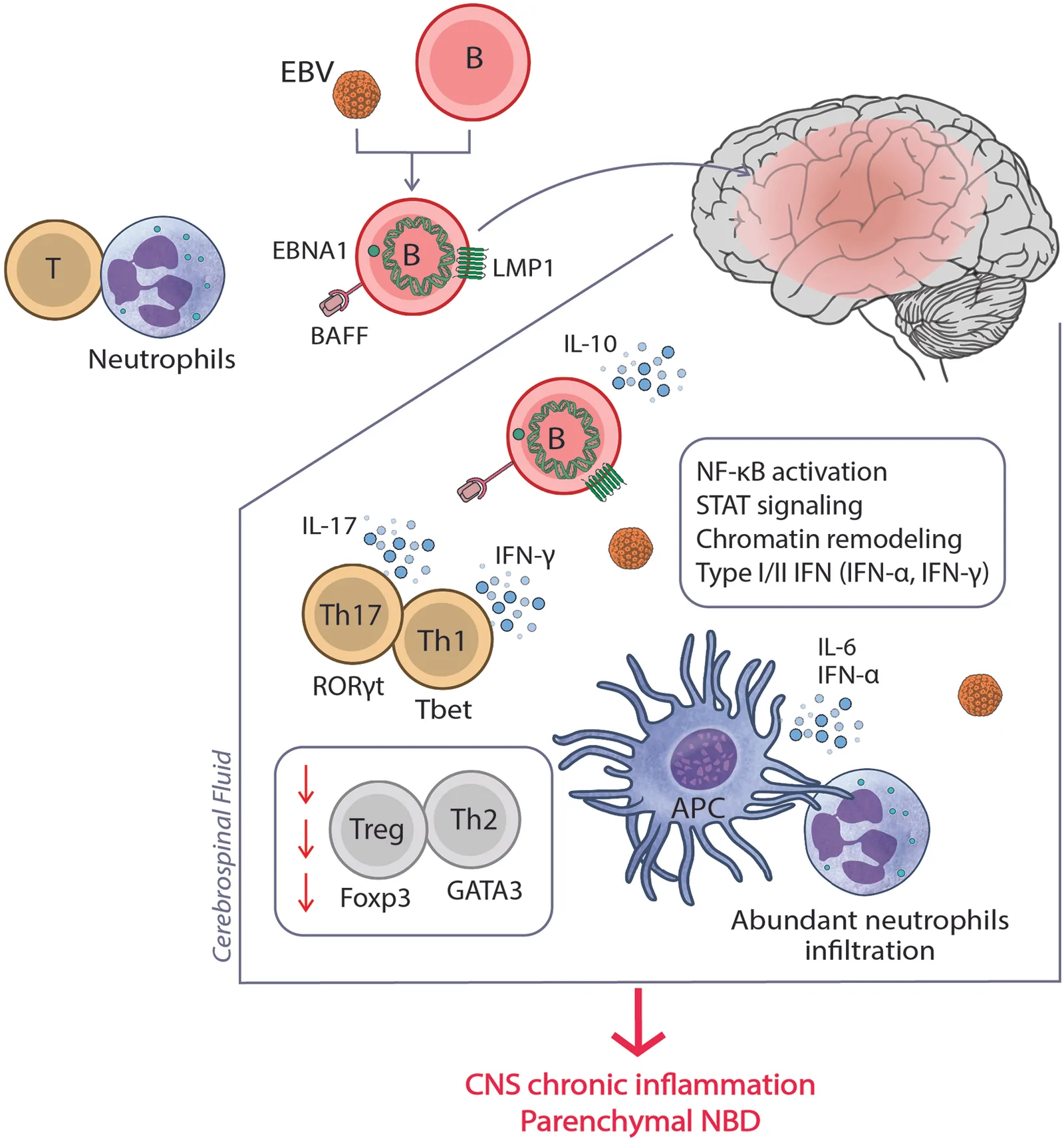

## Introduction

Neuroinflammation represents a heterogeneous group of disorders affecting the central nervous system (CNS), in which both autoimmune mechanisms and infectious triggers have been proposed (1, 2). Among these triggers, neurotropic viruses have received growing interest due to their ability to persist within the CNS, often silently, but sometimes triggering immune responses and neural damage (3). A central question remains: are viral infections causal in these disorders, or merely opportunistic consequences of immune dysfunction?

While the pathogenesis of neuroinflammatory disorders remains incompletely understood, herpesviruses (HHVs) have emerged as potential contributors to neuroinflammation (4). Once within the neural environment, HHVs can establish lifelong latency within neural tissues and may reactivate during periods of immune dysregulation (5, 6). Among the nine known human HHVs, herpes simplex virus HSV-1, HSV-2, varicella zoster virus (VZV), cytomegalovirus (CMV), and Epstein–Barr virus (EBV), are the most extensively studied in the context of neuroinfectious diseases (7–9). A potential link between HHVs and Multiple Sclerosis (MS) has been suspected for decades, particularly for EBV, which has emerged as a compelling environmental factor (10). The severity of primary EBV infection strongly correlates with an increased risk of developing MS (11). It was reported that MS risk increased 32-fold following severe EBV infection, whereas no such increase is seen with other viruses, including CMV. Proposed mechanisms of autoimmunity onset comprise molecular mimicry, bystander damage, and the presence of EBV-infected autoreactive B cells (12). Notably, high titers of Epstein-Barr Nuclear Antigen 1 (EBNA1)-specific antibodies have been associated with MS development, possibly through molecular mimicry between the EBNA1 epitope and the CNS glial cell adhesion molecule GlialCAM, triggering cross-reactive autoimmune responses (13). Beyond its immunogenicity, EBNA1 is essential for viral genome replication and episomal maintenance, while latent membrane protein 1 (LMP1) functions as a major viral oncoprotein that promotes cellular transformation and activates survival and proliferative signaling pathways (14). As central regulators of EBV latency, both proteins contribute to immune modulation and viral persistence, making them particularly relevant targets in studies investigating EBV-associated neuroinflammation. While EBV has dominated research attention, human herpesvirus 6 (HHV-6), once proposed as a potential contributor to MS, has shown inconsistent and non-specific associations with the disease, and its role in MS pathogenesis remains uncertain (15). Beyond neuroinflammation, EBV has been linked to a range of severe complications, including hemophagocytic lymphohistiocytosis and chronic active EBV disease, with recent reports of associated cardiovascular sequelae such as coronary artery aneurysm and thrombosis (16). Furthermore, the virus possesses a well-established oncogenic potential, driving the development of several malignancies, including Burkitt and Hodgkin lymphomas, nasopharyngeal carcinoma, and post-transplant lymphoproliferative disease (17).

Despite growing interest in the viral contribution to MS, data remain scarce in several populations, particularly in North Africa, especially for rare neuroinflammatory disease like neuro-Behçet’s disease (NBD), where the involvement of HHVs has not yet been investigated. NBD is a severe neurological manifestation of Behçet’s disease (BD), the most common form of systemic vasculitis in Tunisia (18), with a distinctive geographical distribution, being far more common in countries along the historic Silk Road than in the rest of the world (19). NBD is observed in approximately 10–30% of patients with BD (20). This disease is regarded as multifactorial, involving genetic predisposition, environmental factors, and microbial triggers (21). Indeed, one of the most longstanding hypotheses is that infectious agents, especially viruses, may act as triggers of systemic inflammation responses in BD (22), a theory initially suggested by Hulusi Behçet himself in 1937. While pathogens are no longer considered the primary cause, they may act as secondary or opportunistic stimuli that activate immune pathways (23). Rather than pointing to a single infectious cause, it is thought that a diverse group of microorganisms sharing common antigens provokes dysregulated immune responses (24). Supporting this notion, HSV-1 DNA, elevated anti-HSV-1 antibody titers, and immune complexes containing HSV-1 antigens have been found in BD patients’ serum (25). Other viruses, including hepatitis C, parvovirus B19, CMV, EBV, and VZV, have also been implicated in BD (24). Notably, recent evidence highlights chronic active EBV infection as a useful biomarker to differentiate BD diagnosis from Behçet-like mucocutaneous syndromes (26). In parallel, immune-mediated mechanisms such as heat shock protein responses, altered cytokine profiles, and T cell hypersensitivity have been proposed as key contributors to BD pathogenesis (24, 27).

Behçet’s disease shows a strong genetic association with HLA-B51, which, through its structural interaction with KIR3DL1/KIR3DS1 and its presentation of MICA-derived peptides, drives an HLA-B51-restricted CD8^+^ T cell response in patients with BD (28, 29). This establishes CD8^+^ T cells as a genetically anchored component of BD immunopathogenesis, in contrast to the predominantly CD4^+^ T cell-centered framework through which EBV’s contribution to MS has been characterized. Our approach in this study focused on CD4-lineage transcription factors (T-bet, GATA3, RORγt, and FOXP3) follows this MS-derived CD4 framework.

Given the growing body of suggestive evidence implicating viruses in BD and the lack of studies on their role in NBD, this research aimed to bridge this critical gap. To address this question, we screened the cerebrospinal fluid (CSF) of NBD patients for neurotropic viruses, using MS as a comparative neuroinflammatory autoimmune disease. In parallel, serum samples from patients were analyzed to assess virus-specific immune response. We further investigated the intrathecal immune response by evaluating gene expression profiles associated with Th1, Th2, Th17, regulatory, pro-inflammatory, and anti-viral pathways. Finally, clinical profiles of NBD patients were compared according to the presence or absence of viral infection.

## Results

### Patients characteristics

The main demographic and clinical characteristics of the study participants are summarized in **Table 1**. Paired blood and CSF samples were obtained from 35 NBD patients and 51 RRMS patients, all at the time of their first clinical relapse. All the participants were treatment-naïve and had not received immunosuppressive or immunomodulatory therapy prior to sampling. While age did not differ significantly between groups (NBD, 38.45 years; RRMS, 35.04 years; *p* = *0.124*, Mann–Whitney test), a significant difference was observed in sex distribution (*p = 0.02*, Fisher’s exact test), with NBD occurring predominantly in men and MS in women in line with previous reports (30, 31). In addition, RRMS patients also exhibited substantially higher intrathecal immunoglobulin synthesis (IgG index = 0.92) than NBD patients (0.46; *p* < 0.0001, Mann–Whitney test). Measures of BBB integrity, assessed using the CSF-to-serum albumin ratio, are represented in **Table 1**.

**Table 1:** Biological and clinical characteristics of patients included in the study.

Disease	NBD	RRMS
Number of patients	35	51
Mean age (sd)	38.45 (± 18.2)	35.04 (± 13.1)
Sex ratio (F/M)	14/21	33/18
IgG index (sd)	0.46 (± 0.29)	0.92 (± 0.19)
CSF/serum albumin ratio (10–3)	5.12 (± 2.05)	4.67 (± 3.13)
Patients in relapse	All	All
Patients under therapy	None	None

### Intrathecal EBV as a hallmark of NBD

To determine whether neurotropic viruses are differentially present in NBD (n=35) and MS (n=51), we first screened patients CSF for a panel of prevalent RNA viruses, including Enterovirus, Measles virus and SARS-CoV-2 (for patients recruited after 2020) by qPCR. No viral RNA was detected in any samples from either cohort. These samples were next screened for the presence of DNA from five common HHVs using multiplex PCR (**Figure 1**). Our results show that no HHV-7 DNA was detected in any CSF samples. VZV showed a prevalence of around 9% in both NBD and MS samples. HSV-1/2 was detected in 23.33% (n=7) of NBD samples compared with 11.43% (n=4) of MS samples. HHV-6 DNA was enriched in NBD CSF samples (15.63%) (n=5) as compared to MS samples (n=1). However, none of these differences reached statistical significance, underscoring their sporadic occurrence in both conditions. In stark contrast, a statistically significant difference was observed for EBV, the DNA of which was present in 25/35 (71.43%) of NBD analyzed samples compared with 18/51 (35.29%) of MS samples (*p = 0.002*, Chi-square test). To ensure the robustness of this finding, all positive and borderline results from the initial multiplex PCR screen were systematically validated by uniplex PCR, confirming the EBV-positive status in all cases and excluding potential false negatives due to assay competition. Furthermore, within the NBD cohort, the prevalence of EBV was significantly higher than that of other herpesviruses, including HSV-1 (*p = 0.0002*), VZV (*p < 0.0001*), HHV-6 (*p < 0.0001*), and HHV-7 (*p < 0.0001*, Fisher’s exact test). These results highlight a strong and specific association between CSF EBV presence and NBD.

**Figure 1:**
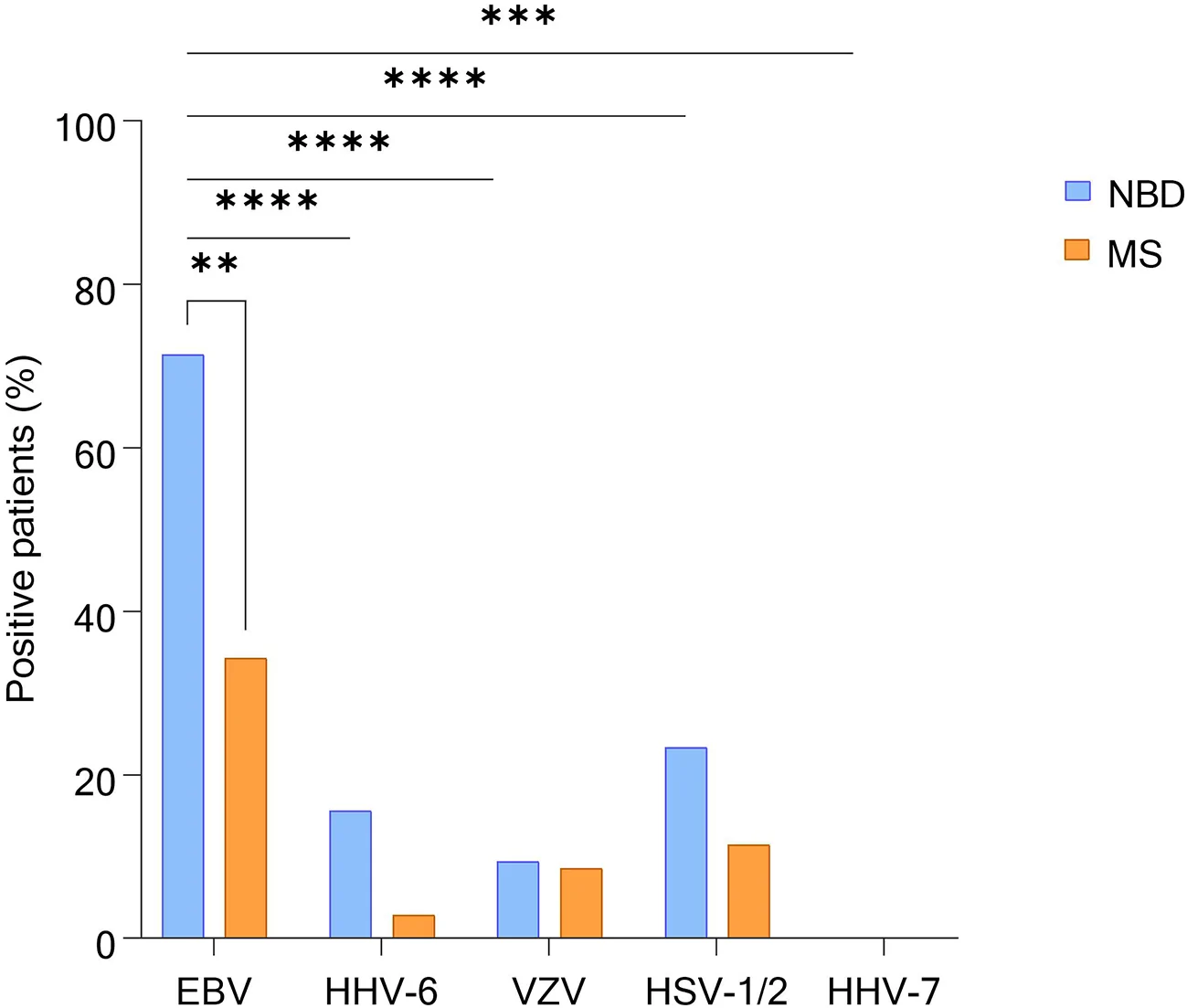
Prevalence of herpesviruses detected in the cerebrospinal fluid of Neuro-Behçet’s disease and multiple sclerosis patients. Histogram showing the frequency of detection of Epstein–Barr virus (EBV), Human Herpesvirus 6 (HHV-6), Varicella-Zoster virus (VZV), Herpes Simplex Virus types 1 and 2 (HSV-1/2), and Human Herpesvirus 7 (HHV-7) in cerebrospinal fluid samples from patients with Neuro-Behçet’s disease (NBD) and multiple sclerosis (MS). ** *p < 0.01*, *** *p < 0.001*, **** *p < 0.0001*.

### CNS presence of EBV DNA is not associated with intrathecal antibody synthesis

We next assessed whether EBV and HSV-1 infection were associated with a local antibody response. IgG antibodies against HSV-1 antigen, latent (EBNA1) and EBV lytic (VCA) antigens were measured in paired CSF and serum samples from NBD MS cohorts.

As expected, systemic anti-EBV IgG antibodies were frequently detected in the serum of most patients, with highly significant titers for both anti-EBNA1 (*p = 0.0006*) and anti-VCA (*p = 0.0151*) antibodies present in NBD and MS samples (**Figure 2**). By contrast, these antibodies were detected at very low levels in CSF samples from both NBD and MS patients. In serum, anti-HSV-1 IgG antibodies were significantly more prevalent in MS patients than in NBD patients (*p < 0.0001*). However, no difference was observed in the secretion of these antibodies in the CSF between the two studied groups.

**Figure 2:**
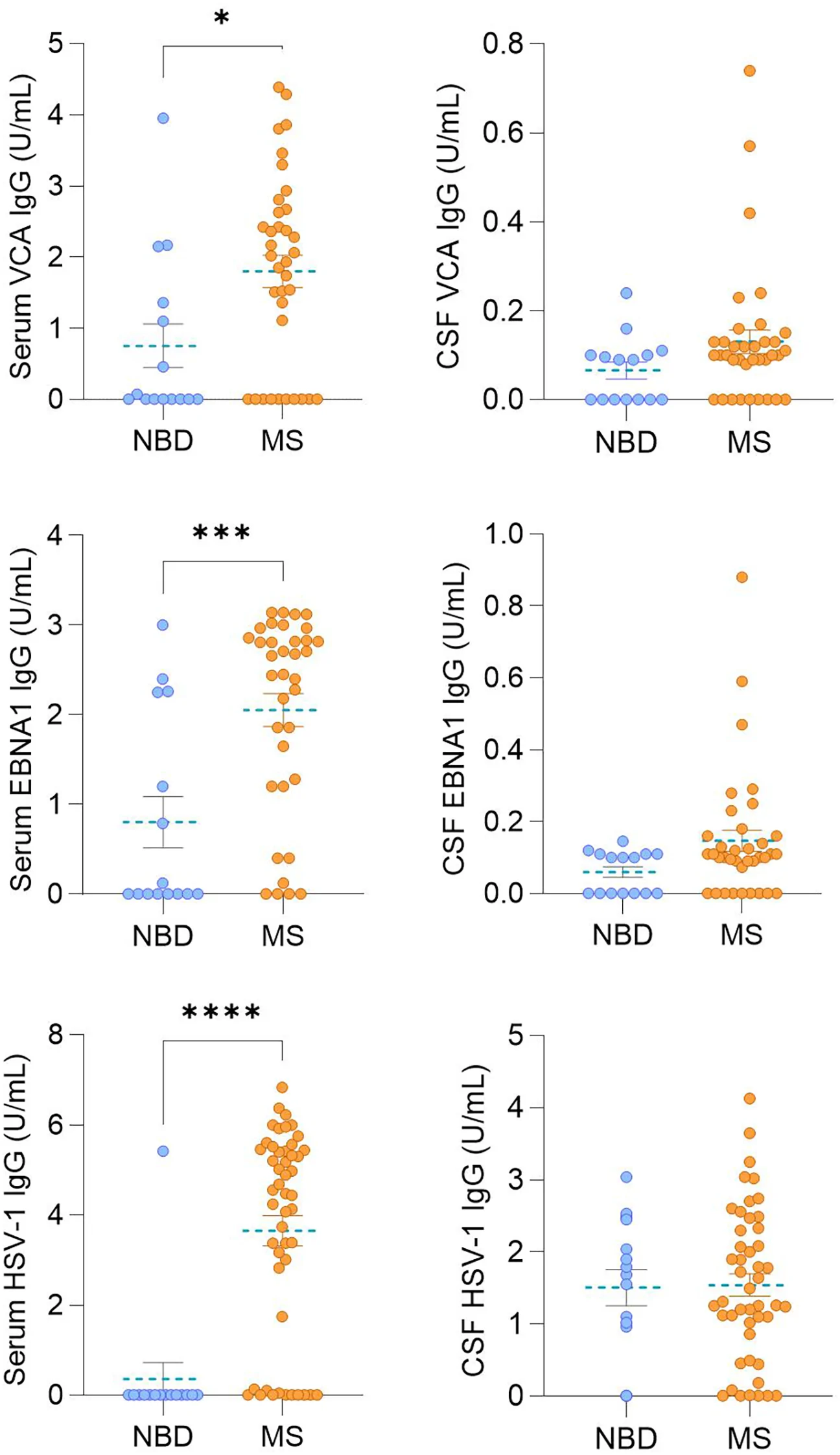
Detection of EBV- and HSV-1-specific antibodies in the serum and cerebrospinal fluid of Neuro-Behçet’s disease and multiple sclerosis patients. Enzyme-linked immunosorbent assay (ELISA) was used to assess antibody responses against Epstein–Barr virus (EBV) viral capsid antigen (VCA), EBV nuclear antigen 1 (EBNA1), and Herpes Simplex Virus type 1 (HSV-1) in paired serum and cerebrospinal fluid samples from patients with Neuro-Behçet’s disease (NBD) and multiple sclerosis (MS). * *p < 0.05*, *** *p < 0.001*, **** *p < 0.0001*.

Together, these findings reveal a dissociation between EBV and HSV-1 DNA detection and intrathecal humoral immunity.

### LMP1 mRNA expression correlates with EBNA1 in the CSF of NBD patients

Cerebrospinal fluid of EBV DNA–positive NBD patients were further analyzed for viral transcriptional profile. Given EBV’s lifelong persistence in latency, we focused on latent viral genes. Among the latter, we selected EBNA1 and LMP1, given their key role in EBV biology. Quantitative real-time PCR (RT-qPCR) was performed using 14 CSF EBV DNA–positive samples.

Our analysis revealed a significant positive correlation between LMP1 and EBNA1 expression levels (Pearson’s r = 0.6372, *p = 0.0142*), indicating a coordinated expression pattern of these latent viral genes in the CNS environment of NBD patients (**Figure 3**).

**Figure 3:**
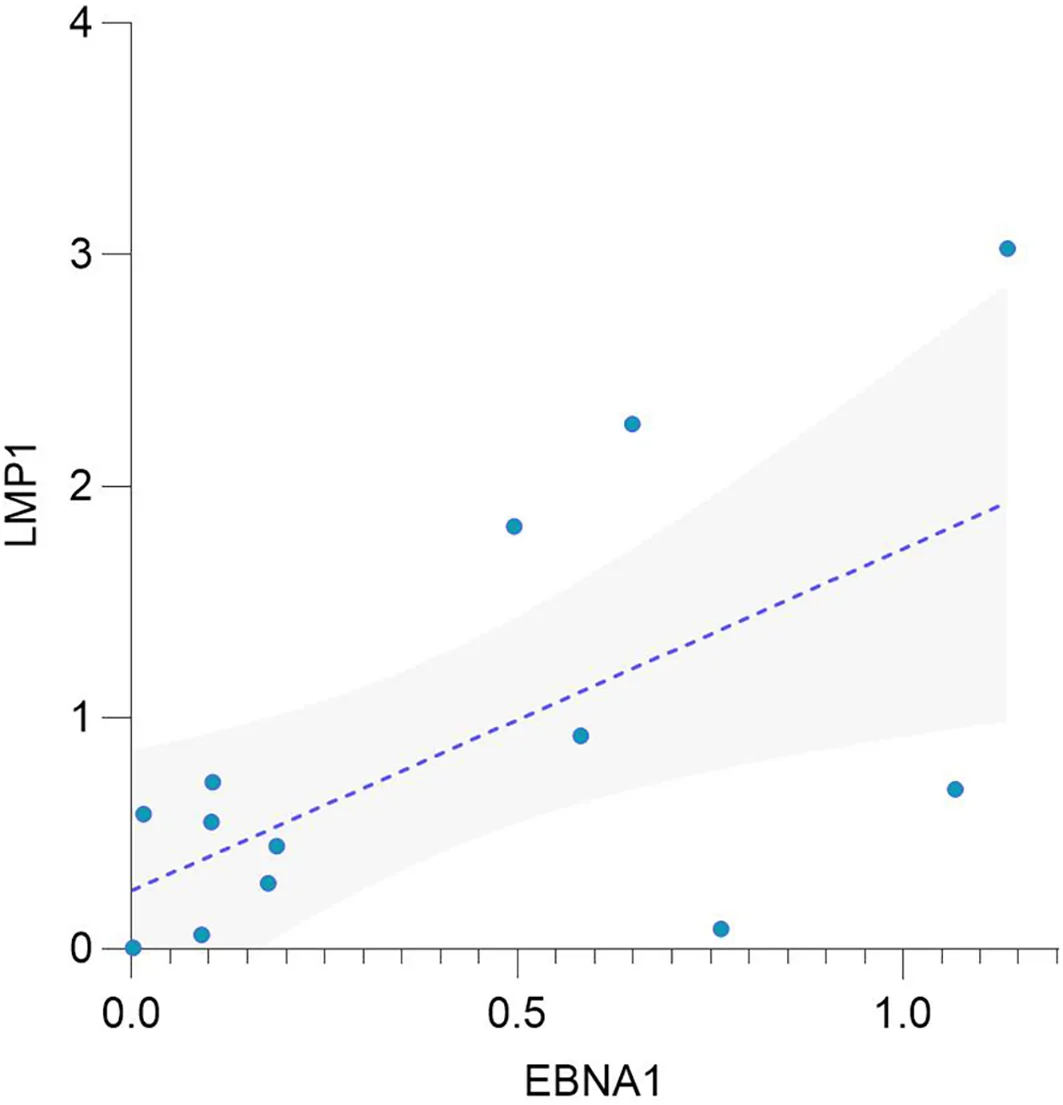
Co-expression of EBV latent genes LMP1 and EBNA1 in EBV-positive Neuro-Behçet’s disease patients. Quantitative PCR (qPCR) analysis of latent membrane protein 1 (LMP1) and Epstein–Barr virus nuclear antigen 1 (EBNA1) expression in samples from EBV-positive Neuro-Behçet’s disease patients.

### Cytokine mRNA expression in NBD depending on EBV infection

Given the central role of the immune response in controlling viral infections and shaping inflammatory processes, we next investigated whether cytokine expression patterns in the CSF of NBD differed according to EBV infection status. For this analysis, patients with NBD were stratified according to EBV infection status into EBV-positive (n=14) and EBV-negative (n=10) groups. A broad panel was quantified by qPCR, including interferon γ (IFN-γ), IL-4, IL-17, IL-10, as well as IL-6, TNF-α, IL-1β, and IFN-α. These mediators represent key pathways involved in T-cell differentiation and regulation, comprising respectively Th1 (T-bet), Th2 (GATA3), Th17 (RORγt), and regulatory T-cell (FOXP3) responses, as well as pro-inflammatory viral signaling networks (**Figure 4**).

**Figure 4:**
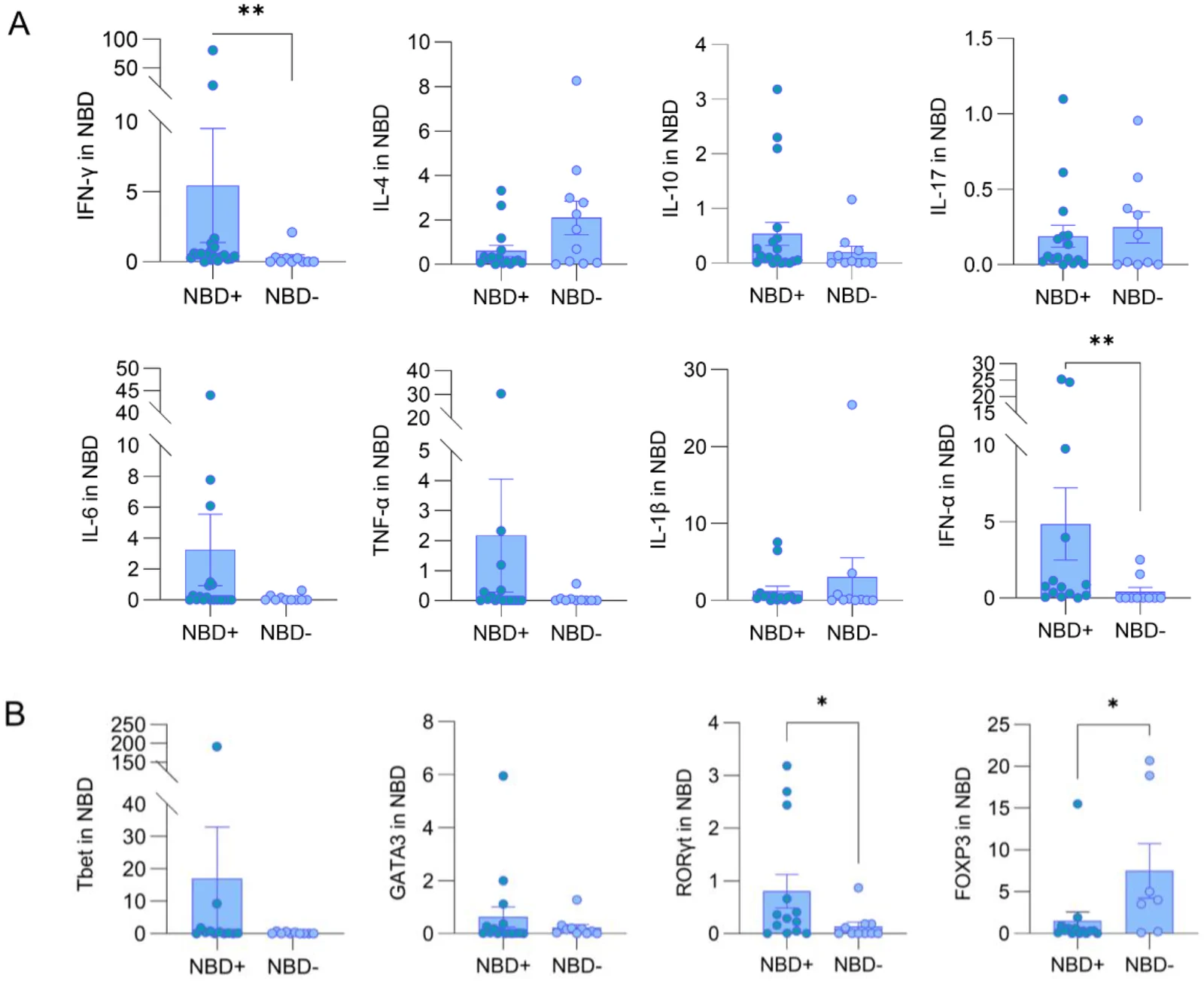
Differential expression of cytokines and transcription factors according to EBV status in the cerebrospinal fluid of Neuro-Behçet’s disease patients. Gene expression analysis by qPCR showing **(A)** cytokine and **(B)** transcription factor expression profiles in the cerebrospinal fluid **(CSF)** of Neuro-Behçet’s disease patients according to EBV DNA detection status. NBD+ denotes patients positive for EBV DNA in the CSF, while NBD− denotes patients negative for EBV DNA in the CSF.

EBV-positive patients exhibited a significantly higher expression of IFN-γ (*p = 0.0083*) than EBV-negative patients, indicating an enhanced Th1 polarization. Consistent with this finding, the Th1-associated transcription factor T-bet tended to be more highly expressed in EBV-positive patients. In contrast, IL-4 expression was lower in EBV-positive samples compared to EBV-negative controls, suggesting a reduced Th2 response. Although GATA3 levels did not show a marked difference, the decrease in IL-4 supports an overall shift away from Th2-associated immune response. FOXP3 expression was significantly reduced in EBV-positive patients (*p = 0.0376*), indicating an imbalance between regulatory and Th17-related markers. IL-17 did not display a significant difference between groups, although a trend toward a significantly increased RORγt expression (*p = 0.0264*) was observed in EBV-positive samples. We also observed a slightly higher expression for IL-6 and TNF-α pro-inflammatory cytokines, and it was significant for IFN-α (*p = 0.0054)* in EBV-positive samples. However, we observed no difference in the expression of IL-1beta between the two studied groups.

### B cells are behind the production of IL-10 in NBD CSF

Among the analyzed cytokines, IL-10 was selected because of its well-established role in the immunomodulation of persistent viral infections, particularly infection with the EBV, where it is considered a key mediator in the regulation of the immune response (**Figure 5**). Flow cytometry analysis of CSF cells from EBV-positive NBD patients showed that IL-10 expression was predominantly localized to CD19^+^ B lymphocytes, with a significantly higher frequency than that observed in CD4^+^ T lymphocytes (*p* < 0.0001).

**Figure 5:**
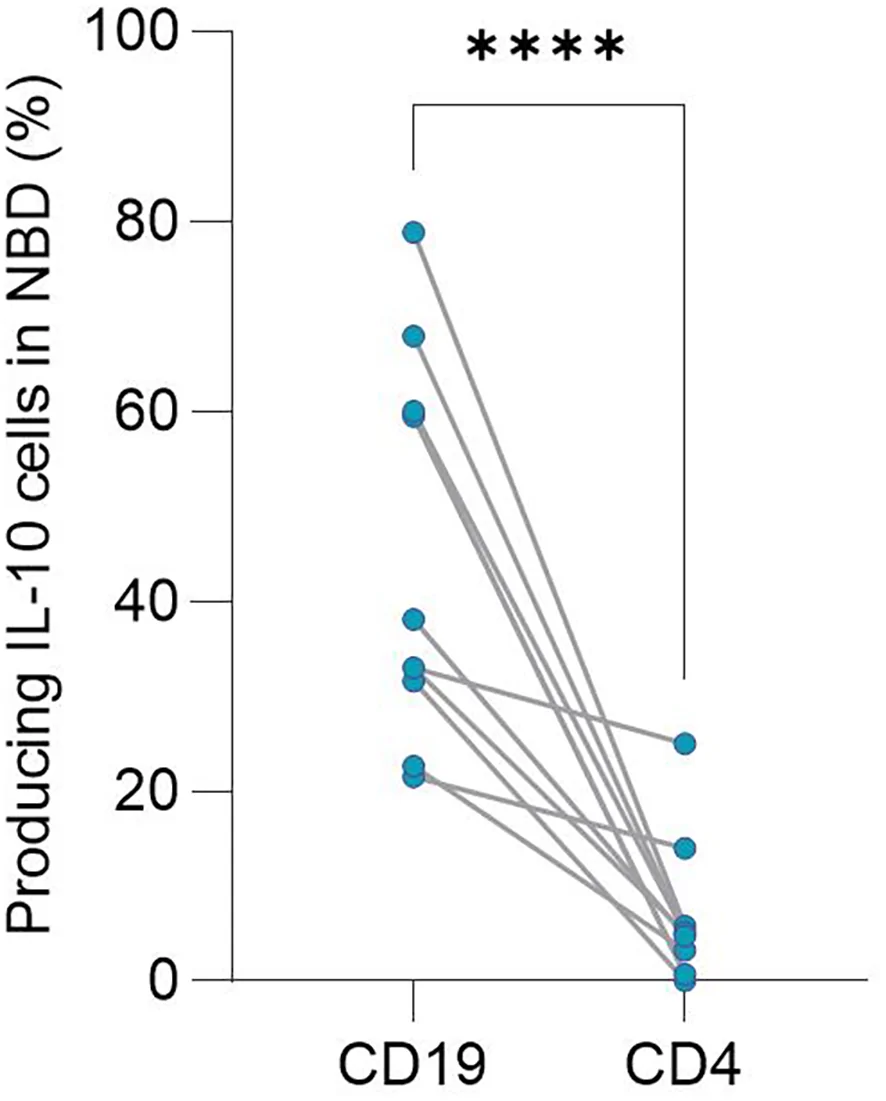
Differential IL-10 expression in B- and T-cell subsets of EBV-positive Neuro-Behçet’s disease patients. Flow cytometric analysis of intracellular IL-10 expression in B- and T-lymphocyte subsets from EBV-positive Neuro-Behçet’s disease patients revealed that B cells constitute the predominant IL-10-expressing cell population.

### Clinical study of NBD patients based on EBV infection

To investigate a potential association between EBV infection and disease manifestations, clinical characteristics of all NBD patients were compared according to EBV detection in the CSF (**Figure 6**). Based on the international criteria for NBD, we systematically characterized the neurological and systemic manifestations in our cohort, stratified by EBV status. All patients with parenchymal involvement are positive to EBV (100%; *p = 0.0008*, Chi-square test), whereas 75% of patients with vascular involvement are negative to EBV (*p < 0.0001*).

**Figure 6:**
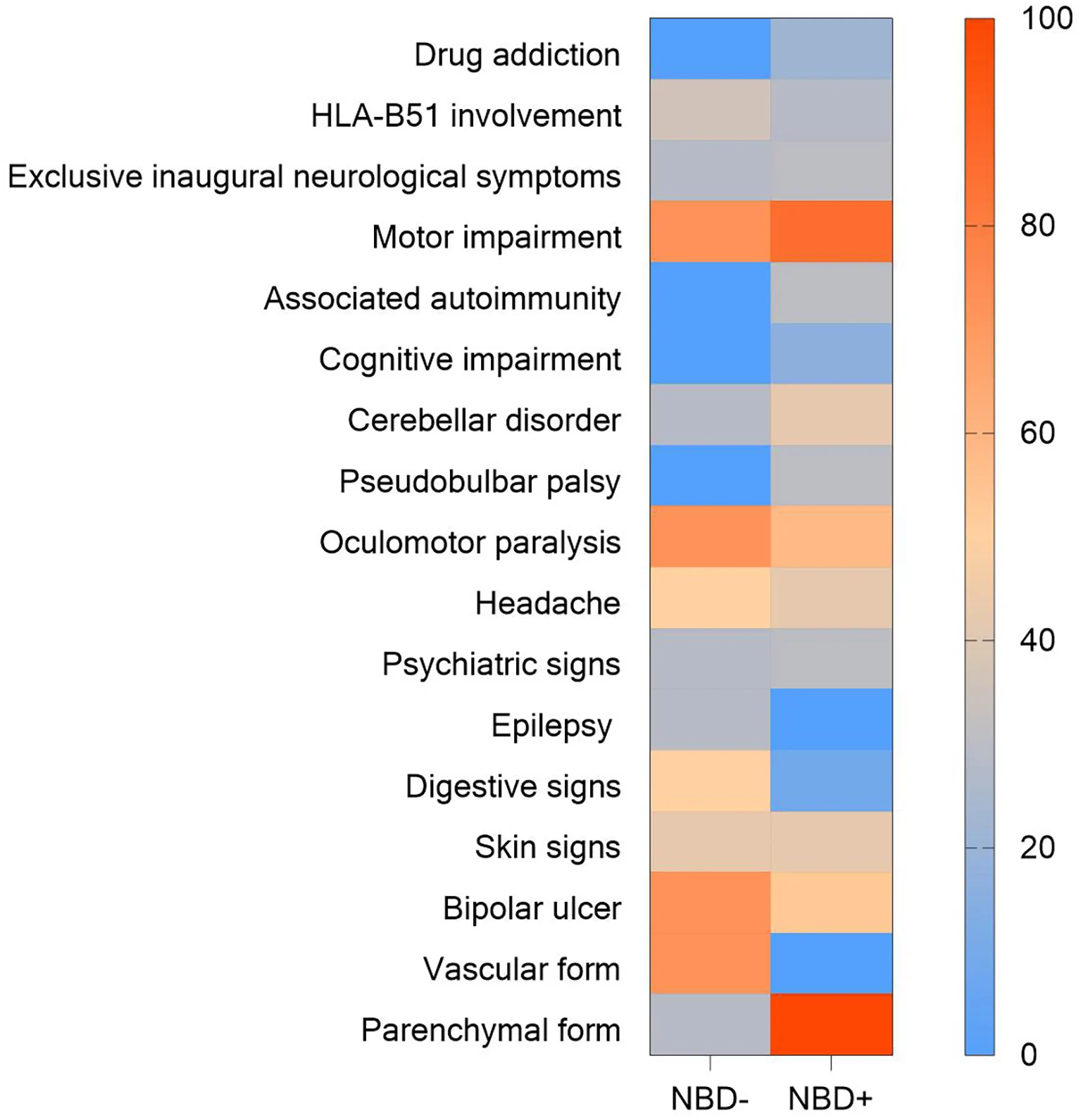
Heatmap of clinical manifestations in Neuro-Behçet’s disease patients according to cerebrospinal fluid EBV DNA status. Heatmap showing the frequency (%) of clinical manifestations in Neuro-Behçet’s disease patients stratified by the presence or absence of Epstein–Barr virus (EBV) DNA in the cerebrospinal fluid (CSF). NBD+ denotes patients positive for EBV DNA in the CSF, whereas NBD− denotes patients negative for EBV DNA in the CSF. Color intensity reflects the relative frequency of each clinical manifestation within each group.

Digestive manifestations were significantly more frequent in EBV-negative patients (50% vs 6.7%, *p = 0.0233*). Epilepsy was also observed only in EBV-negative individuals (25% vs 0%, *p = 0.0346*). Pseudobulbar palsy, cognitive impairment, associated autoimmunity and drug addiction were only reported in EBV-positive patients.

## Discussion

EBV’s role in neuroinflammation is best characterized in MS (32), where seroconversion precedes and increases disease risk, with mechanisms including molecular mimicry and CNS B-cell persistence. In NBD, prior work has been limited to peripheral serology suggesting polyclonal activation rather than an EBV-specific effect, without examining the CSF compartment directly. To our knowledge, this study provides the first systematic comparison of intrathecal herpesvirus detection in NBD identifying a markedly higher prevalence of EBV DNA in the CSF. Strikingly, in our cohort, we found intrathecal EBV DNA in 71.4% of CSF samples from NBD patients, significantly more frequent than in MS (*p = 0.002*), whereas VZV, HHV-6, HHV-7 and HSV-1/2 were detected only sporadically across both groups. These findings identify a strong association between intrathecal EBV DNA and NBD and suggest that EBV may be relevant to the neuroinflammatory environment of NBD.

Few studies have investigated virus serology in NBD. Marta et al. reported significantly higher EBV-VCA IgG in NBD versus BD patients, alongside a parallel, non-significant rise in anti-measles IgG (33). Similarly, Irschick et al. observed EBV, CMV, and HHV8 DNA more frequently in blood samples from patients with BD than in controls, without corresponding differences in plasma IL-4, IL-6, IL-8, or IL-10 levels; however, these findings did not reach statistical significance (34).

In our study, EBV DNA detection was not accompanied by a disease-specific intrathecal anti-EBNA1 or anti-VCA IgG response. Despite higher serum anti-EBV IgG titers in RRMS than NBD, EBV-specific antibodies were undetectable in CSF in both groups. This dissociation between viral DNA and intrathecal humoral responses may reflect the limited sensitivity of antibody-based detection in the CNS or the contribution of cellular rather than humoral mechanisms to EBV control. However, our data do not allow us to determine whether EBV DNA finding represents local viral persistence, reactivation, or the presence of EBV-infected cells within the CSF compartment. Nevertheless, this result is consistent with previous reports that EBV-specific intrathecal antibody responses do not necessarily reflect systemic anti-EBV immunity in MS (34, 35).

We further investigated the EBV-associated immune profile in NBD. EBV positive patients showed a significant positive correlation between LMP1 and EBNA1 mRNA expression (r = 0.6372, *p = 0.0142*) suggestive of a coordinated regulatory program compatible with features of type II latency (36). This coordinated expression may also reflect shared regulation by NF-κB/STAT signaling and inflammation-responsive chromatin remodeling, potentially promoting sustained viral gene expression and a pro-inflammatory intrathecal environment (37–42).

EBV-positive patients also exhibited a distinct inflammatory and immunoregulatory profile. The increase in IFN-γ, combined with a decrease in IL-4, suggests a shift toward a Th1-dominant response rather than a balanced Th1/Th2 response. This may reflect the activity of Th1-type CD4^+^ T cells and/or cytotoxic CD8^+^ T cells, both of which are major sources of IFN-γ during EBV-associated immune responses (37–39). The concomitant increase in IFN-α could indicate activation of innate antiviral pathways in response to the intrathecal presence of EBV (40, 41). Besides, RORγt increased expression, combined with a decrease in FOXP3 expression, further suggests an imbalance in transcriptional regulation between Th17 and Treg cells. Although IL-17 alone did not reach a statistically significant threshold, this transcriptional profile, in the context of increased IL-6 and TNF-α, could indicate an inflammatory environment conducive to Th17 cells rather than overt Th17 cytokine production (48–50). The decreased expression of FOXP3 is also consistent with impaired regulatory activity and with previous observations reporting reduced Treg responses during EBV infection (42).

It is well established that CD8^+^ T cell responses to EBV are predominantly directed against lytic-cycle rather than latent antigens, whereas our study measured the latency-associated transcripts EBNA1 and LMP1; a lytic-phase CD8^+^ response therefore could not be captured by our approach (43, 44). Nonetheless, EBV is increasingly understood to drive autoimmunity through immune dysregulation involving EBV-infected B cells and cognate antigen-recognizing T cells within CNS ectopic lymphoid tissue, a mechanism described for MS (45). This framework offers a plausible, though CD4-biased, model for how intrathecal EBV latency could still promote neuroinflammation in NBD, while underscoring that a complementary assessment of lytic-phase CD8^+^ responses is needed to fully reconcile our findings with the CD8-dominant immunogenetics of BD.

The B-cell compartment represents an additional link between EBV and the inflammatory environment of NBD, which is characterized by elevated intrathecal levels of BAFF, IL-6, and IL-10. In the present study, flow cytometry analysis demonstrated that B lymphocytes were the predominant IL-10-producing population, which is consistent with our previous findings regarding NBD (46). It should be noted that the LMP1 is thought to prime IFN-α production in EBV-infected B cells and to upregulate key immunoregulatory factors, including BAFF, IL-6, and IL-10, further supporting its role in shaping a pro-inflammatory and B cell–activating environment (47, 48). BAFF, previously shown to be overexpressed in NBD skin lesions and CSF (49), can drive expansion of IL-10-producing B cells (50), offering a plausible pathway linking B-cell hyperactivity, already implicated in BD (51), to the CD19^+^-dominant IL-10 signature we observed. The coexistence of gene expressions associated with EBV latency and this B-cell-associated cytokine profile establishes a link between EBV persistence and local B-cell activation in NBD. However, our cross-sectional data do not allow us to determine whether EBV contributes to this inflammatory environment or benefits from it.

Interestingly, EBV status was also associated with clinical phenotype. All EBV-positive patients had parenchymal NBD, whereas EBV-negative patients predominantly presented with vascular form. Digestive involvement and epilepsy were significantly more frequent among EBV-negative patients, while pseudobulbar palsy occurred exclusively in EBV-positive cases. The occurrence of epilepsy exclusively among EBV-negative patients may therefore be related to the predominance of vascular NBD in this group, as cerebral venous thrombosis and cortical infarction have been reported as important risk factors for seizures in NBD (52). Similarly, gastrointestinal involvement has been reported more frequently in non-parenchymal than in parenchymal NBD (53). Thus, the higher frequency of digestive manifestations observed in EBV-negative patients may reflect the predominance of the vascular/non-parenchymal phenotype rather than a direct association with EBV negativity.

Although causality cannot be established, our findings support a hypothetical model linking EBV to parenchymal NBD. In the context of the systemic immune dysregulation characteristic of NBD, Th1/Th17-driven inflammation may favor EBV reactivation in infected B lymphocytes. These cells could subsequently cross the already compromised BBB and enter the CNS, where they may release viral antigens and pro-inflammatory mediators, activate microglia, and promote the recruitment of EBV-specific cytotoxic CD8^+^ T cells. The resulting local inflammatory response, characterized by IFN-γ, TNF-α and IL-6, could contribute to tissue injury and the development of parenchymal lesions. EBV antigens such as EBNA1 may further amplify local inflammation through molecular mimicry with CNS proteins. In this study, EBV DNA detected in the CSF could reflect either local viral persistence/reactivation or the presence of EBV-infected B lymphocytes within the intrathecal compartment. However, the cross-sectional design, limited sample size and restricted virological and immunological profiling preclude conclusions regarding causality or the precise source and biological significance of EBV DNA in the CSF. The significant difference in sex distribution between the NBD and RRMS cohorts may represent a potential confounder of the comparative findings between the two groups. Larger longitudinal and multicenter studies integrating viral, cellular, and functional analyses are needed to determine whether EBV contributes directly to NBD pathogenesis or instead reflects the inflammatory environment. In this context, transcriptomic profiling of EBV-positive NBD samples could further elucidate the molecular mechanisms underlying EBV-associated disease and help identify novel biomarkers of EBV-positive NBD.

## Materials and methods

### Patients and sample collection

From 2017 to 2024, a total of 86 patients (35 NBD and 51 MS) were recruited at the National Institute of Neurology Mongi Ben Hamida (Tunis, Tunisia). All patients were enrolled based on the presence of a first clinical episode of acute inflammatory neurological disease, prior to the initiation of anti-inflammatory and immunosuppressive treatment. The definitive diagnosis of either MS or NBD was confirmed after 1 to 12 months of follow-up. Blood and CSF samples were collected at the time of hospital admission. Due to the limited CSF volume available from NBD patients, the initial cohort screened for viruses was divided into two subgroups, one for RNA viruses, cytokine mRNA and protein expression analyses and the other for flow cytometry allowing complementary characterization of the immune response associated with EBV infection. This stratification optimized the use of limited biological material while allowing a comprehensive characterization of the presence and activity within the CNS.

This study was approved by the Biomedical Ethics Committee (CEBM) of the Institut Pasteur de Tunis (IPT) (2025/15/I/V1), and written informed consent was obtained from all participants before inclusion in the study.

### Clinical characteristics of patients

Clinical characteristics, including systemic symptoms, brain and spinal MRI findings, and CSF laboratory parameters (Immunoglobulin G (IgG) index and oligoclonal IgG band) were retrospectively collected from patient medical records. Patients with relapsing–remitting MS (RRMS) met the revised McDonald diagnostic criteria (54), whereas patients with NBD fulfilled the revised International Consensus Recommendations of NBD (55).

### Viral RNA and DNA extraction from CSF and polymerase chain reaction

Viral RNA and DNA were extracted from 200 µL of CSF using the QIAamp DSP Virus Spin Kit (*Qiagen, Hilden, Germany*), following the manufacturer’s instructions. Nucleic acids were eluted in 50 µL of elution buffer and stored at −30 °C until PCR analysis.

Multiplex PCR was used to screen for HHVs (**Supplementary Table 1**) in 35 patients with NBD and 51 patients with MS. The protocol was adapted and optimized based on the method described by Tanaka et al. (56), with minor modifications including adjustment of viral DNA/RNA concentrations, the addition of dimethyl sulfoxide (DMSO; Sigma-Aldrich, St. Louis, MO, USA) as an adjuvant, and variation of hybridization temperatures. These modifications were validated on positive control samples before final protocol implementation.

To account for potential primer competition in the multiplex format, selected samples were also re-analyzed using uniplex PCR (individual reactions for each viral target) in order to confirm ambiguous or unexpected results.

PCR amplification was carried out using an Applied Biosystems Veriti Thermal Cycler with 40 cycles for all primer sets. Amplification products were separated by electrophoresis on a 2.5% agarose gel (Invitrogen, Carlsbad, CA, USA) and visualized using a nucleic acid stain (GelRed^®^, Biotium, Fremont, CA, USA).

### Measurement of serum and CSF IgG antibodies

Serum and CSF samples from 15 NBD and 51 MS patients were assayed in duplicate for IgG antibodies against EBV viral capsid antigen (VCA) and nuclear antigen 1 (EBNA1), as well as HSV-1, measles and rubella viruses, using commercial enzyme-linked immunosorbent assays (Platelia™ EBV-VCA IgG, Platelia™ EBNA1 IgG, Platelia™ Measles IgG, Platelia™ Rubella IgG; Bio-Rad) according to the manufacturer’s protocols.

For the EBV assays, samples and controls were diluted 1:21 and incubated for 20 min at 21-25 °C in microplate wells coated with either purified EBV-VCA or recombinant EBNA1 or HSV-1. For the measles and rubella assays, samples were diluted 1:101 and 1:21, respectively, and incubated for 45 min (measles) or 1 hour (rubella) at 37 °C in wells coated with the corresponding viral antigens.

For all assays, after the initial incubation and a wash step, wells were incubated with a horseradish peroxidase-conjugated anti-human IgG secondary antibody (20 min at 21-25 °C for EBV; 45 min or 1 hour at 37 °C for measles and rubella, respectively). Reactions were developed with tetramethylbenzidine (TMB) and stopped with acid solution. Absorbance was read at 450 nm. For EBV and measles, an immune status ratio (ISR) was calculated by normalizing sample absorbance to a calibrator cut-off value, with ISR ≥ 1.10 (EBV) or >1.2 (measles) considered positive. For rubella, concentrations (IU/ml) were determined from a standard curve, with results ≥ 15 IU/ml considered positive.

### RNA extraction from CSF and conventional RT-PCR

For the qPCR analysis, total RNA from CSF cell pellet, lysed in RLT buffer supplemented with 1% betamercaptoethannol, was extracted from up to 1 × 10^6^ cells using the RNeasy Mini Kit (Qiagen). Genomic DNA was eliminated through an on-column DNase digestion step, performed for 15 minutes at room temperature using the RNase-Free DNase Set (Qiagen). RNA was eluted in 30µL of nuclease-free water and used immediately for complementary DNA (cDNA) synthesis with the High-Capacity cDNA Reverse Transcription Kit (Applied Biosystems), according to the manufacturer’s instructions.

### Quantification by qPCR

To screen for the presence of neurotropic RNA viruses, cDNA generated from CSF RNA was first analyzed by quantitative real-time PCR (qPCR) using probe-based assays targeting Measles virus, SARS-CoV-2, and Enterovirus. All qPCR assays were performed in technical duplicate. These analyses were performed at the Laboratory of Clinical Virology of IPT. Amplification reactions were performed using TaqMan-based assays on the ABI QuantStudio Real-Time PCR System (Thermo Fisher Scientific, Waltham, MA), according to the manufacturer’s instructions. Positive RNA controls for measles were obtained from CDC (Atlanta, GA, USA). For SARS-Cov-2, the detection was performed according to the World Health Organization (WHO)-approved protocol published by Corman et al. (57) and detecting specific sequences in the envelope (E), nucleocapsid protein (N), and RNA-dependent RNA polymerase (RdRp) genes (**Supplementary Table 2**). Viral detection was determined based on amplification curves and cycle threshold (Ct) values, with samples showing no amplification or Ct values comparable to negative controls considered negative.

The same cDNA samples were subsequently used to quantify the expression of cytokines and transcription factors specific to Th1 (Tbet), Th2 (GATA-3), Th17 (RORyT), and Treg (FOXP3) cells, as well as EBV latency-associated (EBNA1 and LMP1) genes in the CSF. Gene-specific primers were designed using NCBI Primer-BLAST (**Supplementary Table 3**) and synthesized by RAN BioLinks. qPCR reactions were performed using SYBR Green Master Mix (Thermo Fisher Scientific). Relative gene expression was calculated using the 2−ΔCt method, with GAPDH used as the housekeeping gene for normalization.

### Intracellular detection of IL-10

Freshly isolated CSF cells from 10 EBV-positive patients with NBD were analyzed individually by flow cytometry. For each patient, cells were divided into stimulated and unstimulated aliquots, with the latter serving as controls. Stimulated cells were incubated for six hours in the presence of PMA (50 ng/mL, Sigma-Aldrich), ionomycin (500 ng/mL, Sigma-Aldrich) and GolgiPlug (BD Biosciences, Paris, France). Membrane staining was then performed using anti-CD19 (HIB19) and anti-CD4 (RPA-T4) antibodies, conjugated with fluorescein isothiocyanate (FITC) and allophycocyanin (APC) respectively (BD Biosciences, Paris, France). Cells were subsequently fixed and permeabilized in accordance with the recommendations of the Cytofix/Cytoperm™ kit (BD Biosciences, Paris, France). Intracellular IL-10 was detected by incubation with a phycoerythrin (PE)-conjugated anti-IL-10 antibody (clone JES3-9D7) for 1 hour at +4 °C in the dark. A total of 100, 000 events were collected for each sample. Flow cytometric analysis was performed by sequential gating of lymphocyte populations based on forward and side scatter (FSC/SSC), followed by the selection of single cells and live cells. CD19+ and CD4+ cell populations were then identified, and the level of IL-10 expression was determined as the percentage of IL-10+ cells within each population. Thus, the denominator for IL-10 frequencies was the total number of live CD19+ or CD4+ cells, respectively. The data were processed and analyzed using Kaluza software.

### Statistics

GraphPad Prism (version 10.4.2 for Windows; GraphPad Software, San Diego, CA, USA) was used for all graphing, calculations, and statistical analyses. Comparisons between groups were performed using the Mann–Whitney U test, while paired nonparametric data were analyzed using the Wilcoxon signed-rank test. Categorical variables describing patient characteristics were analyzed using contingency tables and the appropriate Chi-square or Fisher’s exact test, depending on expected frequencies. No adjustment for multiple comparisons was applied. A p-value ≤ 0.05 was considered statistically significant.

## Data Availability

The datasets presented in this study can be found in online repositories. The names of the repository/repositories and accession number(s) can be found in the article/**Supplementary Material**.
