# Supplementary material for: Epstein–Barr virus shapes intrathecal Th1/Th17 immunity and parenchymal involvement in neuro-Behçet’s disease
Source: Front Immunol. 2026 Oct 8;17:1921667. doi: 10.3389/fimmu.2026.1921667 (PMC13687393; doi:10.3389/fimmu.2026.1921667)
Supplement: Supplementary file 1 [file DataSheet1.docx]

**Supplementary Table 1:** Primer sequences of the target genes in PCR and real time PCR

| **Target Gene** | **Sequence 5’-3’** | **Type** | **Annealing temperature** | **Product size** |
| --- | --- | --- | --- | --- |
| VZV | TTTGGGTTCTCTTGGCTGTT | Forward | 62°C | 161bp |
| VZV | TCCATTATCCGCTACATCTGAA | Reverse | 58°C |  |
| HSV-1/2 | CGAGATGCCTTCAGCAGAGT | Forward | 60°C | 292bp |
| HSV-1/2 | CCCTTAAAGTCCTCCAGCAA | Reverse | 62°C |  |
| EBV | GACTCCATCTTGGCTGTGA | Forward | 62°C | 229bp |
| EBV | TGATTTCTGCTCTGACAACCT | Reverse | 60°C |  |
| HHV-6 | TGATTCACACTTAAAGGAGACGGC | Forward | 66°C | 183bp |
| HHV-6 | CCTCTGGAGCCTGACCTGTGAT | Reverse | 68°C |  |
| HHV-7 | GCCCGTTTTCGGAAATATTGGAGAGAT | Forward | 68°C | 347bp |
| HHV-7 | ACGCACGAGACGCACTTTTCTTAAACA | Reverse | 69°C |  |
| Touchdown PCR has been carried out to enhance amplification specificity for the multiplex PCR, with an initial annealing temperature of 70°C decreasing stepwise to 58°C over 35 cycles. | | | | |

**Supplementary Table 2:** Primer sequences of the target RNA viruses in real time PCR

| **Target Gene** | **Sequence 5’-3’** | **Type** | **Annealing/**  **acquisition temp.** | **Product size** | **Reference** |
| --- | --- | --- | --- | --- | --- |
| SARS-CoV-2  (ORF1b-nsp14) | TGGGGYTTTACRGGTAACCT | Forward | 60°C | 132bp | https://www.who.int/docs/default-source/coronaviruse/whoinhouseassays.pdf |
| SARS-CoV-2  (ORF1b-nsp14) | AACRCGCTTAACAAAGCACTC | Reverse |  |  |  |
| SARS-CoV-2  (ORF1b-nsp14) | FAM-TAGTTGTGATGCWATCATGACTAG-TAMRA | Probe |  |  |  |
| SARS-CoV-2  (E gene/E_Sarbeco) | TAATCAGACAAGGAACTGATTA | Forward | 60°C | 110bp |  |
| SARS-CoV-2  (E gene/E_Sarbeco) | CGAAGGTGTGACTTCCATG | Reverse |  |  |  |
| SARS-CoV-2  (E gene/E_Sarbeco) | FAM-GCAAATTGTGCAATTTGCGG-  TAMRA | Probe |  |  |  |
| Enterovirus (5'-UTR) | CACTCGGATGGCC | RT | 42°C | 156 bp | [(Rezig et al. 2004)](https://paperpile.com/c/hHIIjR/Symb) |
| Enterovirus (5'-UTR) | CTCCGGCCCCTGAATGCG | Forward |  |  |  |
| Enterovirus (5'-UTR) | ATTGTCACCATAAGCAGCCA | Reverse |  |  |  |
| Measles (N gene) | TGGCATCTGAACTCGGTATCAC | Forward | 60 °C | 75bp | [(Hummel et al. 2006)](https://paperpile.com/c/hHIIjR/XAmX) |
| Measles (N gene) | TGTCCTCAGTAGTATGCATTGCAA | Reverse |  |  |  |
| Measles (N gene) | FAM-CCGAGGATGCAAGGCTTGTTTCAG A BHQ | Probe |  |  |  |

| **Target Gene** | **Sequence 5’-3’** | **Type** | **Product size** |
| --- | --- | --- | --- |
| IFN-γ | TTTGGGTTCTCTTGGCTGTT | Forward | 103bp |
| IFN-γ | TCCATTATCCGCTACATCTGAA | Reverse |  |
| T-bet | AGGATTCCGGGAGAACTTTGAG | Forward | 81bp |
| T-bet | AATTGACAGTTGGGTCCAGGC | Reverse |  |
| IL-4 | CACGGACACAAGTGCGATA | Forward | 102bp |
| Il-4 | GATGTCTGTTACGGTCAACTCG | Reverse |  |
| GATA3 | CAAAATGAACGGACAGAACCG | Forward | 66bp |
| GATA3 | GCTCTCCTGGCTGCAGACA | Reverse |  |
| IL-10 | CGAGATGCCTTCAGCAGAGT | Forward | 95bp |
| IL-10 | CCCTTAAAGTCCTCCAGCAA | Reverse |  |
| FOXP3 | AGCTGGAGTTCCGCAAGAAAC | Forward | 101bp |
| FOXP3 | TGTTCGTCCATCCTCCTTTCC | Reverse |  |
| IL-17 | ACCAATCCCAAAAGGTCCTC | Forward | 176bp |
| IL-17 | TGGATGGGGACAGAGTTCAT | Reverse |  |
| RORγt | AATGACCAGATTGTGCTTCTCAAAG | Forward | 82bp |
| RORγt | GGTTGTCAGCATTGTAGGCCC | Reverse |  |
| IL-6 | ACAGACAGCCACTCACCTC | Forward | 136bp |
| IL-6 | TTTTCTGCCAGTGCC TCTTT | Reverse |  |
| TNF-α | CCAGAGGGAAGAGTTCCCCA | Forward | 91bp |
| TNF-α | AGGCTTGTCACTCGGGGTTC | Reverse |  |
| IL-1β | AATCTGTACCTGTCCTGCGTGTT | Forward | 78bp |
| IL-1β | TGGGTAATTTTTGGGATCTACACTCT | Reverse |  |
| IFN-α | GACTCCATCTTGGCTGTGA | Forward | 103bp |
| IFN-α | TGATTTCTGCTCTGACAACCT | Reverse |  |
| LMP1 | CCTACTGATGATCACCCTCCTC | Forward | 93bp |
| LMP1 | AAGCACCCGAAGATGAACAGC | Reverse |  |
| EBNA1 | TGATTCACACTTAAAGGAGACGGC | Forward | 95bp |
| EBNA1 | CCTCTGGAGCCTGACCTGTGAT | Reverse |  |
| GAPDH | TGGAAGGACTCATGACCACAGTC | Forward | 83bp |
| GAPDH | ATCACGCCACAGTTTCCCGG | Reverse |  |

**Supplementary Table 3:** Primer sequences of the target genes in real time PCR
